# Supplementary material for: Non-reciprocal and non-Newtonian mechanical metamaterials
Source: Nat Commun. 2023 Aug 8;14:4778. doi: 10.1038/s41467-023-40493-6 (PMC10409733; doi:10.1038/s41467-023-40493-6)
Supplement: Supplementary file 1 — Supplementary Information [file 41467_2023_40493_MOESM1_ESM.pdf]

Supplementary Information

Non-reciprocal and Non-Newtonian Mechanical Metamaterials

Lianchao Wang[1,2], Julio A. Iglesias Martínez[2], Gwenn Ulliac[2], Bing Wang[1,*], Vincent Laude[2], Muamer Kadic[2,*]

[1]National Key Laboratory of Science and Technology on Advanced Composites in Special Environments, Harbin Institute of Technology, Harbin, 150001, PR China
[2]Université Franche-Comté, CNRS, Institut FEMTO-ST, Besançon 25000, France

Corresponding author. Email: wangbing86@hit.edu.cn
Corresponding author. Email: muamer.kadic@univ-fcomte.fr

1 Experimental frequency response of the tilted cantilevers

We designed and fabricated the samples to obtain a clear genuine first-order resonant frequency for the tilted cantilevers, as shown in Fig. S1a. The geometry parameters of the cantilevers of this sample are the same as the counterparts of the 2D unit cell that are demonstrated in Section 2 in the main text. The experimental setup illustrated in Fig. S1b is employed to evaluate the vibrations of the samples under varying external source frequencies. It is worth noting that in this setup the bottom of the samples, or substrate, is fixed to the piezoelectric source but the top of the samples is left free. Other parameters are the same as for the setup of section 2 in the main text.

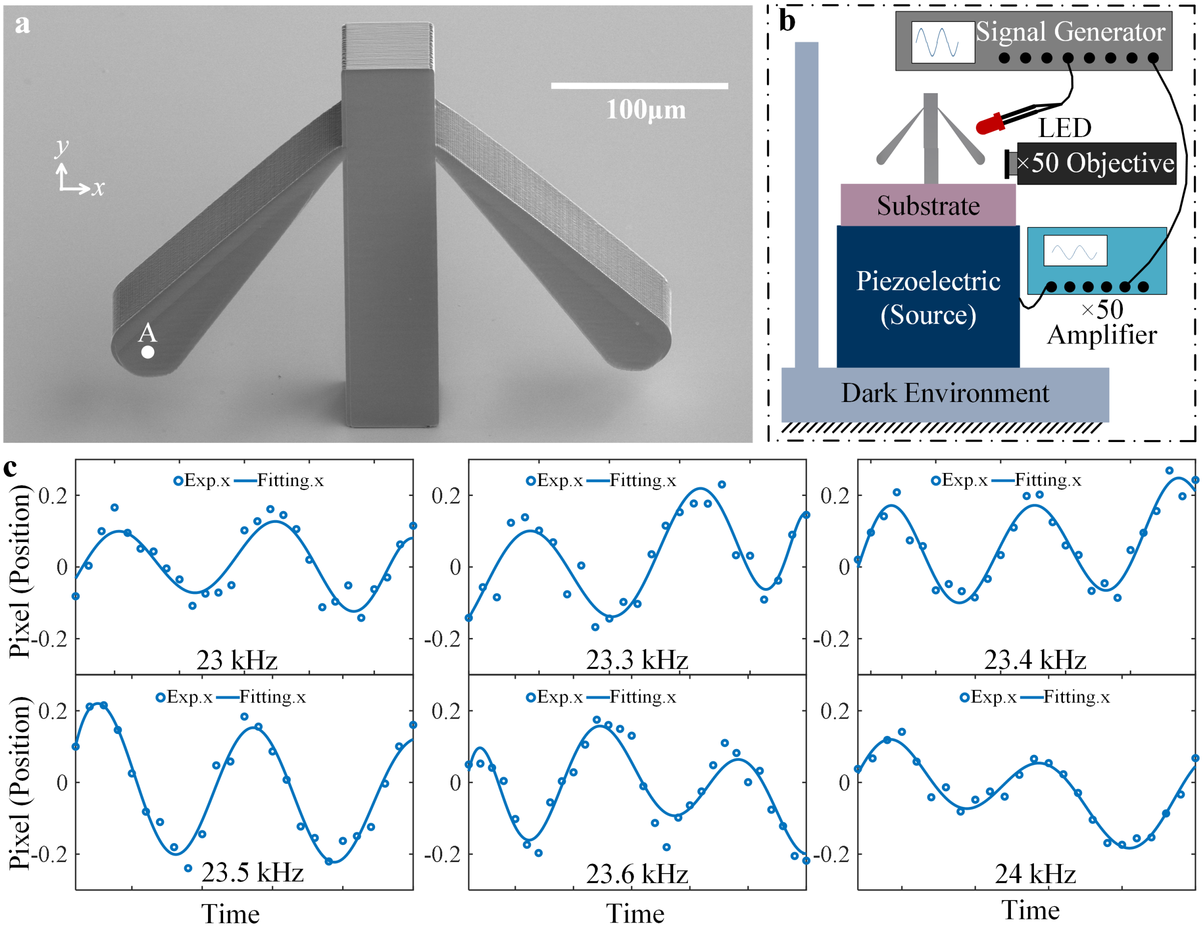

Figure S1. Experimental frequency sweep for the tilted cantilevers. a A scanning electron microscope (SEM) image of the sample used in the experimental frequency sweep in shown. **b** The instrumentation setup for experimental frequency sweep allows us for in-plane vibration monitoring as a function of time. **c** The *x*-displacement of point A at the free end of the cantilevers is plotted versus time for different driving frequencies.

The image tracking algorithm of MATLAB is used to extract the displacement of point A (see Fig. S1a) in the *x*-direction as a function of time, as shown in Fig. S1c. In principle, the external excitation (the piezoelectric source) vibrates only in the *y*-direction, so the displacement in the *x*-direction is introduced by the elastic vibration of the cantilever. When the external excitation frequency is 23.5 kHz, the displacement of the cantilevers in the *x* direction reaches its maximum value, from which we conclude that the first-order resonance frequency of the cantilever is 23.5 kHz.

2 Influence of the stiffness of the main beam on the resonance of the cantilevers

Simulations were conducted to understand why the elastic vibrations of the tilted cantilevers can be excited when the external stimuli come from source 2, as the measurements in Fig. S2a suggest. As in the experimental setup, the FEM model and the experimental samples studied in Section 2 of the main text have identical geometry parameters, all degrees of freedom at the bottom of the structure are fixed, and an external harmonic force at the resonant frequency of the cantilever is applied at the top of the structure, i.e., source 2. It is worth noting that Young's modulus for all parts in the unit cell is set to 4 GPa except for the main beam (the light blue region in Fig. S2a). The Young's modulus of the main beam is either considered as 4 GPa or 16 GPa respectively.

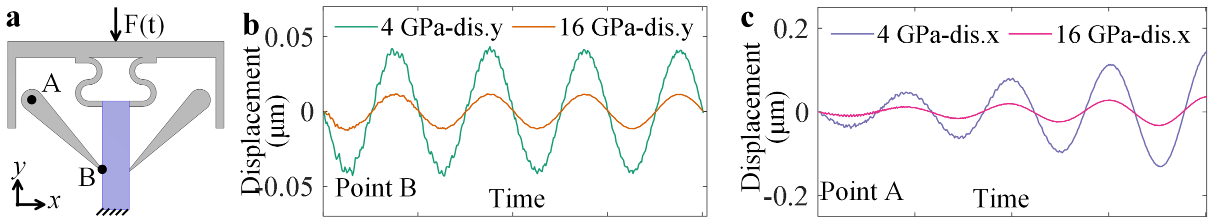

Figure S2. Influence of the stiffness of the main beam on the resonance of the cantilevers. a The setting details for FEM simulations are depicted. **b** The y-displacement of the fixed end of the cantilevers (i.e., point B) is shown versus time for 2 different values of the stiffness of the main beam. **c** The x-displacement of the free end of the cantilevers (i.e., point A) is shown versus time for both values of the stiffness of the main beam.

The displacements of the fixed end (point B) and the free end (point A) of the cantilever in the y- and the x-directions are shown versus time in Fig. S2b and c, respectively. In principle, when external stimuli act on the structure from source 2, the fixed end of the cantilevers would remain idle in the y-direction if the main beam were an ideal rigid body. In fact, the main beam is elastic and source 2 induces its elastic deformation. This type of longitudinal elastic deformation decreases with the value of Young's modulus of the main beam, as observed in the simulation results reported in Fig. S2b.

As a matter of fact, the amplitude of the displacement of point A in the x-direction decreases with the decrease of the amplitude of the y-displacement of point B (i.e., the increase of Young's modulus of the main beam form 4 GPa to 16 GPa), as Fig. S2c shows. It is clear that the vibrations of the tilted cantilevers after a finite time remain close to zero if Young's modulus of the main beam is very large. Then it is easy to conclude that source 2 should not introduce elastic vibrations of the cantilevers in the case that the main body can be considered a rigid body.

3 Experimental study on the mechanical response of the metamaterials

To evaluate the non-Newtonian mechanical performance of the considered metamaterials, the following quasi-static compression and impact test of the metamaterials were performed, as shown in Fig. S3. Three types of samples were designed and fabricated, named sample A-C and illustrated in Fig. S3d-*i*, e-*i*, and f-*i*, respectively. The geometrical parameters for sample A are: $l = 38.2$ mm, $l_2 = 4.3$ mm, $t_3 = 14$mm, $l_3 = t_4 = r_1 = 5$ mm, $l_5 = 98.8$ mm, $l_b = 10$ mm, $t_1 = 1.5$ mm, $t_2 = 1.5$ mm, $t_5 = 16$ mm, $t_b = 4$ mm, $r = 3$ mm, $d = 1$ mm, $h_b = 9$ mm, $l_1 = 22.5$ mm, and $\theta = 45°$. Sample B is identical to sample A but without the fixed boundaries. Sample C is identical to sample B but without the tilted cantilevers (i.e., the subsystems).

First, the INSTRON 3344 mechanical measurement machine was employed for the quasi-static compression test of sample A. The static compression deformation and corresponding force versus compression displacement curve are shown in Fig. S3a and b. It is clear from Fig. S3a that the subsystems (i.e., the tilted cantilevers) are not contacting with the fixed boundary during the whole compression process. In other words, in this case, the static mechanical properties of the metamaterial only depend on the S-shaped beams (corresponding to the main spring K of the toy model). The maximum reaction force value is 5.5 N, as demonstrated in Fig. S3b.

In order to assess the dynamic behavior of the metamaterials, we built the experimental setup for the impact test shown in Fig. S3c. The setup consists of an acceleration sensor, an impactor, a signal processor, and a fixed frame. The sampling frequency of the acceleration sensor is 2 KHz. The mass of the impactor is 4.6 kg and it can free-fall (the height is set to 60 cm) along the vertical frames. The acceleration data of the impactor is generated by the signal processor directly. The acceleration of the impactor is used to obtain the reaction force (multiplying it by the mass of the impactor) of the metamaterials.

The impact response for sample A is illustrated in Fig. S3d. Force as a function of time includes 4 peaks, as shown in Fig.S3d-*ii*. As a remark, the last 3 peaks are caused by bounces of the impactor. It is also worth noting that the first peak corresponds to the collapse of the metamaterial, at which time the main central beam (corresponding to the main mass M in the toy model) contacts the bottom of the metamaterial, as demonstrated in the insert of Fig. S3d-*ii*. Before the collapse, the tilted cantilevers are deformed (see Fig. S3d-*iiii*) and the mechanical response is shown in Fig. S3d-*iii*. There also a peak that can be observed when the force reaches 570 N, after which the force decreases due to the nonlinear mechanical response of the cantilevers under large deformations. The simulation results show a very similar trend in the nonlinear mechanical response, as presented in Fig. 3 of the main text. Eventually, the force increases dramatically because of the occurrence of the collapse of the whole structure. Significantly, and as compared to the static situation, the mechanical response of the metamaterial approximately increases 100 times (from 5.5 N to 570 N) in the dynamic case, which is the signature of the non-Newtonian behavior.

To investigate the cause of the formation of the non-Newtonian properties of the metamaterial, impact tests for samples B and C were carried out experimentally, and consistent results were obtained and are shown in Fig. S3e and f, respectively. It is clear that both samples B and C have impact responses similar to sample A. However, the peak force (before collapse) of these samples is different: 404 N for sample B (see Fig. S3e-*iii*) and 357 N for sample C (see Fig. S3f-*iii*). The difference in the peak forces for samples A and B (570 N − 404 N = 166 N) is an indication of the reaction of the fixed boundaries. Similarly, the difference in the peak forces for samples B and C (404 N − 357 N = 47 N) expresses the response of the subsystems (the tilted cantilevers). Furthermore, the unequal peak forces for samples A and C (570 N − 357 N = 213 N) proves the influence of the subsystems and the fixed boundaries. As a whole, these observations are consistent with the outcome of the toy model, i.e. that the non-Newtonian behavior is introduced by two factors, one being the resonances of the subsystems and the other being the influence of the fixed boundaries.

For completeness, it should be realized there are some differences between the simulation results of

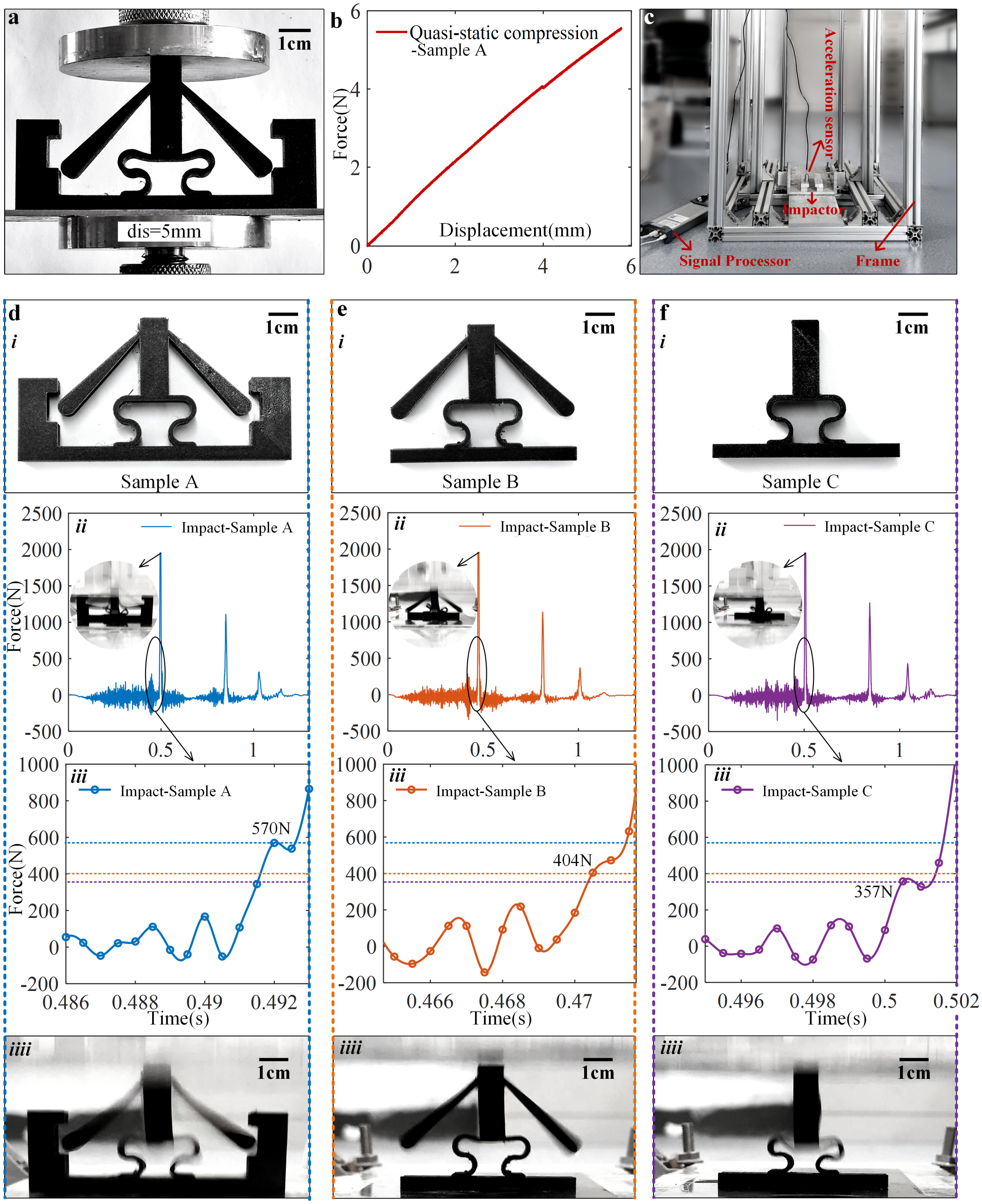

Figure S3. **Experimental validation of the non-Newtonian mechanical behaviors of the considered metamaterial. a** Quasi-static deformation of the metamaterial (sample A, the compression displacement of this moment is 5 mm). **b** The corresponding force versus displacement curve of sample A. **c** Experimental setup for the impact tests. **d-f** The photos of samples A-C and corresponding impact responses.

Fig. 3 in the main text and the experimental results of this section. It is not easy in practice to closely reproduce experimentally the numerical results because of the following issues. First, the elastoplasticity

of the raw material was ignored in the simulations, since we wanted to highlight the operation of the structure. However, elastoplasticity influences the experimental mechanical response, especially for large compressive deformations. Second, the fracture of the parent material is also not taken into consideration in the numerical model. In fact, we initially tried to use PLA as the raw material to print the samples. Nevertheless, the tilted cantilevers broke during the experimental impact tests. Third, in the experiment, we need a larger rectangular opening hole on the fixed boundary to fix the free end of the cantilever beam, because larger rectangular opening holes provide greater tolerance for experimental error. For example, due to experimental errors (off-axis impact) or manufacturing errors (mass or density of the cantilever beams on both sides are not exactly the same), the impact test may cause a slight asymmetric response of the left and right cantilever beams. The larger rectangular opening holes extend the length of step-A in the displacement-force curves in Fig. 3a of the main text. Fourth, in numerical simulations, the impact velocity was kept constant during the impact process. However, in experiments, the velocity of the impactor actually decreases with time during the impact process.

4 Simulation of satellite docking

The satellite docking model was built to explore the potential application of the proposed metamaterials in the aerospace industry. The model includes two satellites and the proposed metamaterial working as a connector between them, as depicted in Fig. S4a. The geometry parameters of the connector are the same as for model *i* of the main text and the distance between per-layer tilted cantilevers is 10 mm. The Young's modulus, Poisson's ratio, and density of the connector are 4 GPa, 0.43, and 1110 kg/m^3, respectively. The counterparts of the satellites are 4000 GPa (that very large value is simply chosen so that the elastic deformation of the satellites can be ignored during the docking process), 0.43, and 1750 kg/m^3. Free tetrahedral elements are used to mesh the whole model and a finer mesh is used in areas including complicated shapes to improve the accuracy of calculations. Between the connector and satellite 2, identical boundary pairs with continuity properties are defined. For the other contact pairs, i.e., the pairs between boundaries and tilted cantilevers, the pairs between the bottom surface of satellite 1 and the top surface of the connector, and the pairs between two satellites, a penalty formulation with a factor of 0.95 is employed. Moreover, all degrees of freedom of satellite 2 and the lateral boundaries are fixed, and an initial velocity (0.3 m/s or 1.5 m/s for these two different cases) is given for satellite 1. The time-dependent module of COMSOL with 8 10^{-5} s time-step is used to simulate the entire docking process.

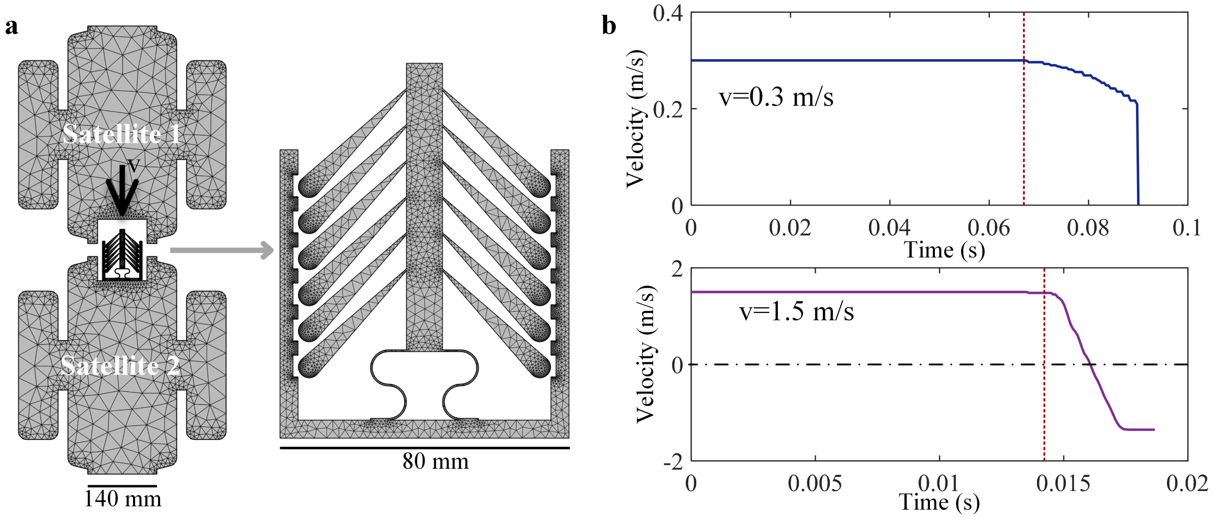

Figure S4. Simulation details for the satellite docking model. a The simulation settings and meshes are shown. **b** The velocity of satellite 1 versus time is platted. From the times marked with dotted lines, the metamaterial operates.

The satellite docking process with varying initial velocities has been demonstrated in Fig. 5 of the main text, and the corresponding animation is available in the supplementary video. Both of them indicate that satellite 1 is able to reach satellite 2 when it has a small initial velocity (0.3 m/s), i.e., that the satellite docking process completes as expected. In contrast, when the impact velocity is large (1.5 m/s) satellite 1 is not in a position to connect with satellite 2 before their relative velocity reduces to 0 m/s, owing to the connector metamaterial showing non-Newtonian mechanical properties so that a lot of kinetic energy of the satellite 1 has been absorbed via the elastic deformations of the tilted cantilevers. The velocity of satellite 1 versus times curves illustrated in Fig. S4b also supports the above conclusion. As can be seen in Fig. S4b, satellite 1 and the connector contact each other from the red dotted line. In the case of a small relative velocity, the velocity of satellite 1 decreases tardily. At the end of the docking process, it goes down to 0 m/s sharply due to the connection between these two satellites. Nevertheless, under the circumstance of a higher impact velocity, the reduction of the velocity of satellite 1 to 0 m/s is dramatic. Its acceleration is then reversed thanks to the release of the elastic energy stored in the connector, thus

avoiding the risk of the two satellites colliding and damaging each other. All in all, the non-Newtonian mechanical properties of the proposed metamaterials show huge application prospects in the aerospace industry, the transportation industry, and soft robotics as well.
