# Supplementary material for: Non-reciprocal and non-Newtonian mechanical metamaterials
Source: Nat Commun. 2023 Aug 8;14:4778. doi: 10.1038/s41467-023-40493-6 (PMC10409733; doi:10.1038/s41467-023-40493-6)
Supplement: Supplementary file 3 — Description of Additional Supplementary Files [file 41467_2023_40493_MOESM3_ESM.pdf]

Description of Additional Supplementary Files

Supplementary Movie 1
Description:

Section 1:
The external excitations applied as source 1 with 10KHz;
The external excitations applied as source 2 with 10KHz;
The external excitations applied as source 1 with 23.5KHz;
The external excitations applied as source 2 with 23.5KHz.

Section 2:
Model i under source 1 with 0.002m/s compression speed;
Model ii under source 1 with 2m/s compression speed;
Model iii under source 1 with 2m/s stretching speed;
Experimental tests of model I under source 1 with 0.002m/s and 2m/s compression speed;
Model I under source 2 with 0.002m/s or 2m/s compression speed (identical response);
Metamaterials (2×2 model i units) under source 1 with 2m/s compression speed.

Section 3:
Satellite docking process with an initial speed of 0.3m/s;
Satellite docking process with an initial speed of 1.5m/s.
