# Supplementary material for: Non-reciprocal and non-Newtonian mechanical metamaterials
Source: Nat Commun. 2023 Aug 8;14:4778. doi: 10.1038/s41467-023-40493-6 (PMC10409733; doi:10.1038/s41467-023-40493-6)
Supplement: Supplementary file 5 — Source Data [file 41467_2023_40493_MOESM5_ESM.zip › Source data/cover letter.docx]

This file includes the source data for the following article,

Non-reciprocal and Non-Newtonian Mechanical Metamaterials

Lianchao Wang^1,2^, Julio A. Iglesias Martnez^2^, Gwenn Ulliac^2^, Bing Wang^1,*^, Vincent Laude^2^, Muamer Kadic^2,*^

^1^ National Key Laboratory of Science and Technology on Advanced Composites in Special Environments, Harbin Institute of Technology, Harbin, 150001, PR China

^2^ Université de Franche-Comté, CNRS, Institut FEMTO-ST, Besançon, 25000, France

^*^wangbing86@hit.edu.cn; [muamer.kadic@univ-fcomte.fr](mailto:muamer.kadic@univ-fcomte.fr)

Journal: *Nature Communications (NCOMMS-23-10939)*

1. **In the source data.xlsx**

--sheet1: the data for Fig. 2g

--sheet2: the data for Fig. 2i

--sheet3: the data for Fig. 3a

--sheet4: the data for Fig. 3c

--sheet5: the data for Fig. 3d

--sheet6: the data for Fig. S1c

--sheet7: the data for Fig. S2b

--sheet8: the data for Fig. S2c

--sheet9: the data for Fig. S3b, Fig. S3d-ii, Fig. S3e-ii, Fig. S3f-ii

--sheet10: the data for Fig. S4b
